# Dynamic m6A epitranscriptomic remodeling coordinates host transcriptional reprogramming during MPXV infection

**DOI:** 10.1186/s12964-026-03090-w

**Published:** 2026-07-18

**Authors:** Ruixia Tang, Jindi Huang, Wenhai Yu, Shuaiyao Lu, Jienan Zhou, Kaiyun Ding, Junbin Wang, Cong Tang, Xiaoqing Fu, Tingfu Du, Qiangming Sun

**Affiliations:** 1 Institute of Medical Biology, Chinese Academy of Medical Sciences & Peking Union Medical College, Kunming, Yunnan Province China; 2 State Key Laboratory of Respiratory Health and Multimorbidity, Beijing, China; 3 Key Laboratory of Pathogen Infection Prevention and Control (Peking Union Medical College), Ministry of Education, Beijing, China; 4 National Kunming High-level Biosafety Primate Research Center, Institute of Medical Biology, Chinese Academy of Medical Sciences and Peking Union Medical College, Kunming, China; 5 Yunnan Provincial Key Laboratory of Vector-borne Diseases Control and Research, Kunming, China; 6 Yunnan Center for Disease Control and Prevention, Kunming, China

**Keywords:** MPXV, m6A, Transcriptome, Virus-host interaction, Immune evasion

## Abstract

**Background:**

The global emergence of monkeypox virus (MPXV) highlights the urgent need for a deeper understanding of host–pathogen interactions. Although transcriptional responses to MPXV infection have been characterized, the role of epitranscriptomic regulation particularly N6‑methyladenosine (m6A) modification remains largely unexplored.

**Methods:**

We performed an integrated analysis of time‑series transcriptomic and m6A methylome profiles using whole blood samples collected from MPXV‑infected rhesus macaques at 7, 14, and 21 days post‑infection, with distinct animals used at each time point. Host gene expression and m6A modification dynamics were examined over the course of infection. Differential expression and differential m6A modification analyses were conducted, followed by integrative pathway and immune cell signature profiling.

**Results:**

MPXV infection induced sustained host reprogramming, characterized by suppression of immune pathways and activation of metabolic processes. A global increase in m6A modifications was observed, accompanied by upregulation of the methyltransferase METTL3 and downregulation of demethylases (FTO, ALKBH5) and readers (YTHDF1-3). Knockdown of METTL3 or YTHDF2 reduced viral replication, suggesting a proviral role for this regulatory circuit. Integrative analysis identified 38 genes with coordinated changes in both transcription and m6A modification across all three time points. Focusing on literature-curated pathogenic pathways, we further identified 11 dual-regulated host factors. Notably, DNAJB1 was the only gene shared between these two independent selection strategies. m6A peaks near transcription start sites and within 5′UTR positively correlated with gene expression, whereas coding region modifications showed weak negative correlations. Immune lineage signatures showed gradual declines in T cell, NK, and monocyte/macrophage signatures with a progressive increase in B cell signatures. Cross‑dataset comparison confirmed core m6A regulatory trends despite heterogeneity across tissues and viral strains.

**Conclusions:**

This study reveals m6A epitranscriptomic remodeling as a key correlate of the host response to MPXV infection and nominates DNAJB1 alongside the other 10 dual‑regulated genes as candidate host factors for further mechanistic investigation.

**Supplementary Information:**

The online version contains supplementary material available at https://doi.org/10.1186/s12964-026-03090-w.

## Introduction

Mpox, caused by MPXV, represents a re-emerging zoonotic disease with growing public health implications. MPXV is a large, enveloped double-stranded DNA virus of the Orthopoxvirus genus, distinguished by a ~ 197 kb genome that encodes a complete cytoplasmic replication and transcription apparatus [1, 2]. This capacity for autonomous replication within the host cytoplasm, independent of nuclear machinery, renders MPXV a valuable model for investigating host interactions of large DNA viruses. Its coding repertoire of nearly 200 proteins establishes the molecular foundation for self-sufficient replication while simultaneously deploying numerous immunomodulatory factors that functionally antagonize interferon and cytokine signaling pathways, thereby subverting host antiviral defenses [3–5].

The 2022 multinational outbreak was driven primarily by the MPXV Clade IIb lineage, which demonstrated enhanced human-to-human transmissibility [4, 6, 7]. Despite this clinical urgency, the molecular mechanisms by which MPXV systemically reprograms host cellular networks to establish a productive infection remain poorly defined [8–11]. A critical knowledge gap exists in understanding how the virus establishes a permissive intracellular environment, balancing resource utilization for replication while simultaneously evading immune detection. Elucidating these mechanisms is paramount for moving beyond supportive care and developing targeted antiviral strategies.

m6A is the most prevalent internal modification in eukaryotic mRNAs, functioning as a central regulator of gene expression homeostasis and stress responses [12, 13]. Its dynamic deposition, removal, and recognition catalyzed by “writer” (METTL3/METTL14/WTAP), “eraser” (FTO, ALKBH5), and “reader” (YTHDF/DC families) proteins, respectively—govern nearly all aspects of RNA metabolism [14]. In viral infections, m6A plays a complex and context-dependent role. On the one hand, it can modulate the expression of host immune-related transcripts, such as type I interferon, thereby fine-tuning the innate immune response [15, 16]. On the other hand, m6A modifications are also deposited on viral RNAs across diverse viral families, encompassing both RNA viruses (e.g., HIV [17], HCV [18]) and DNA viruses (e.g., KSHV [19, 20]). Notably, viruses like KSHV actively hijack the m6A machinery, altering the activity or localization of its components to remodel the host epitranscriptome in favor of persistent infection [21–23]. These findings position the m6A regulatory network as a crucial battleground in virus-host interactions.

While m6A is established in other viral infections, its role in MPXV infection remains unknown. We hypothesize that MPXV hijacks the host m6A epitranscriptomic network to reprogram the transcriptome, promoting viral replication and immune evasion. To test this, we performed integrated transcriptomic and m6A-methylome profiling of whole blood from MPXV-infected macaques. This study aims to: [1] map m6A epitranscriptomic dynamics during infection; [2] decipher remodeling mechanisms, focusing on m6A machinery regulation; and [3] identify m6A-regulated host genes and pathways that facilitate viral pathogenesis. Our work reveals a new dimension of MPXV–host interplay and identifies potential epitranscriptomic biomarkers for therapeutic intervention.

## Methods and materials

### Experimental animal model and sample collection

All animal procedures were conducted in accordance with institutional guidelines and approved by the Institute of Medical Biology, Chinese Academy of Medical Sciences (IMBCAMS, Approval No. DWSP202312012). Twelve healthy adult rhesus macaques (4–8 years old, 4.5–6.5 kg) from IMBCAMS were randomly assigned into four groups based on sex and body weight: three control animals and nine infected animals. The infected animals were further divided into three time point groups (*n* = 3 per group), designated for euthanasia at 7, 14, and 21 days post-infection (dpi). The three control animals were maintained alongside the infected groups and euthanized together at 21 dpi. The experimental groups received an intravenous injection of 1 × 10⁷ TCID₅₀ MPXV clade IIb, while the control group received an equal volume of sterile PBS. Whole blood samples were collected at the time of euthanasia for subsequent transcriptomic and m6A methylome analyses. Thus, distinct animals were used at each time point, and no repeated measurements were taken from the same individual. All procedures were performed in IMBCAMS’s ABSL-3 facility.

### Cell lines and MPXV infection

Five human cell lines were used in this study: Huh7 (hepatocellular carcinoma, FuHeng #FH0873), A549 (lung adenocarcinoma, ATCC #CCL-185), HUVEC (human umbilical vein endothelial cells, ATCC #CRL-1730), CaCo2 (colorectal adenocarcinoma, ATCC #HTB-37), and THP-1 (acute monocytic leukemia, ATCC #TIB-202). Huh7 and A549 in Dulbecco’s Modified Eagle Medium (DMEM, Gibco); HUVEC in endothelial cell medium (ECM, ScienCell); CaCo2 in Minimum Essential Medium (MEM, Gibco); and THP-1 in RPMI-1640 medium (Gibco). All media were supplemented with 10% fetal bovine serum (FBS, Gibco), 100 U/mL penicillin, and 100 µg/mL streptomycin. THP-1 cells were maintained in suspension at a density of 2–5 × 10^5 cells/mL. Cells were cultured at 37 °C in a humidified atmosphere with 5% CO2.

For MPXV infection, adherent cells (Huh7, A549, HUVEC, CaCo2) were seeded in 6-well plates at a density of 5 × 10^5 cells per well and cultured overnight. THP-1 cells were seeded at a density of 1 × 10^6 cells per well in 6-well plates. The culture medium was then replaced with fresh medium containing MPXV (clade IIb, MOI = 0.1). And cells were incubated for 48 h. Mock-infected cells received an equivalent volume of PBS and were processed in parallel. All experiments involving live MPXV were conducted in a biosafety level 3 facility.

### RNA extraction and quality control

Total RNA was extracted from whole-blood samples using TRIzol reagent (Invitrogen, Catalog No. 10296028CN) per the manufacturer’s protocol. RNA concentration/purity was assessed via NanoDrop 1000 (A260/A280 ≥ 1.8, A260/A230 ≥ 2.0), and integrity via Agilent 2100 Bioanalyzer (RNA Integrity Number (RIN) ≥ 8.0). Only qualified RNA was used for subsequent sequencing.

### Transcriptome sequencing (RNA-seq) and data analysis

RNA-seq was performed by CloudSeq Inc. (Shanghai, China). rRNA was removed using GenSeq^®^ rRNA Removal Kit, and libraries were constructed with GenSeq^®^ Directional RNA Library Prep Kit: RNA was fragmented to ~ 300 nt, first/second-strand cDNA synthesized, end-repaired, dA-tailed, adapter-ligated, PCR-amplified, and purified. Libraries were sequenced on an DNBSEQ-T7 platform in paired-end 150 bp mode, with each library sequenced to a depth of approximately 40 million reads. Three biological replicates were analyzed per time point.

Raw reads were subjected to quality control and adaptor trimming using Fastp software (v0.23.4) [24]. High-quality reads were aligned to the Macaca mulatta reference genome (Ensembl Rhesus 10x) via HISAT2 (v2.2.1) [25], and gene raw counts were obtained using featureCount (v2.0.6) [26]. Data normalization, fold change (FC) calculation, and P-value estimation were performed with edgeR (v4.0.16) [27]. Differentially expressed genes (DEGs) were identified using thresholds of P-value < 0.05 and |log₂FC| ≥ 1.0. Gene Ontology (GO) and Kyoto Encyclopedia of Genes and Genomes (KEGG) pathway enrichment analyses were conducted using clusterProfiler (v4.0.5) [28] with a significance threshold of FDR < 0.05. Gene Set Enrichment Analysis (GSEA) was performed using clusterProfiler, with all genes preranked by log₂FC, against the MSigDB hallmark gene sets [29]. Multiple testing correction was implemented using the Benjamini–Hochberg method. The overlaps of gene sets were visualized using the VennDiagram package (v1.7.3) [30].

To identify temporal expression patterns across the three time points (7, 14, and 21 days post-infection), we performed clustering analysis on all genes with complete expression data (log₂-transformed FPKM values). The optimal number of clusters (k) was determined using two complementary methods: the elbow method (within-cluster sum of squares, WSS) and silhouette analysis [31]. Genes were then assigned to distinct clusters using k-means clustering. Expression trajectories were visualized by plotting mean expression levels with 95% confidence intervals for each cluster.

### Correlation analysis with viral load

Spearman rank correlation analysis was performed to assess the relationship between viral load (log₁₀ copies/mL) and molecular features, including global m6A levels, expression of m6A regulatory genes, persistently modified genes, and core candidate genes. Uninfected controls were assigned a viral load of 0. Multiple testing was corrected using the Benjamini‑Hochberg method (FDR < 0.05).

### m6A Methylation sequencing (MeRIP-seq) and data analysis

MeRIP-seq was performed by CloudSeq Inc. (Shanghai, China). Total RNA was fragmented to ~ 200 nt, and synthetic spike-in controls (positive controls containing m6A modification and negative controls without modification) were added. Immunoprecipitation was performed using the GenSeq^®^ m6A MeRIP Kit (antibody-coupled Protein A/G beads), and the captured RNA was eluted and purified. Libraries for immunoprecipitated (IP) and input samples were constructed with the GenSeq^®^ Low Input Whole RNA Library Prep Kit, qualified by an Agilent 2100 Bioanalyzer, and sequenced on a DNBSEQ-T7 platform in paired-end 150 bp mode (approximately 40 million reads per library). Library sizes were normalized using the total read count normalization method. Three biological replicates were analyzed per time point.

Raw reads were subjected to quality control (Q30 ≥ 80%) and trimmed of adaptors and low-quality sequences using cutadapt (v1.9.3) [32]. Clean reads were aligned to the Macaca mulatta reference genome (Ensembl Rhesus 10x) using Hisat2 (v2.0.4) [33]. m6A-enriched peaks were identified using MACS2 [34] by comparing the IP sample to its corresponding input control. Differential methylation regions between time points were detected using diffReps (v1.55.6) [35] with a threshold of P-value ≤ 1e-5 and absolute fold change (|log₂FC|) ≥ 2. Genes harboring at least one differential methylation region were defined as differentially modified genes (DMGs) and subsequently subjected to GO and KEGG pathway enrichment analyses using clusterProfiler (v4.0.5) with a significance threshold of FDR < 0.05.

Genomic region definitions for peak annotation were based on the rhesus macaque reference genome rheMac10 (GCA_003339765.3) with NCBI RefSeq annotations. The 5′UTR was defined as the segment from the transcription start site to the start codon; the start codon and stop codon were each extended by ± 100 bp; and the 3′UTR was defined as the region from the stop codon to the polyadenylation site. Metagene analysis was performed using MetaPlotR (v1.0.2) to generate normalized peak density profiles across the 5′UTR, CDS, and 3′UTR. For motif discovery, 101-bp sequences centered at peak summits (± 50 bp) were extracted, and de novo motif identification was performed with DREME [36] (E-value ≤ 0.05). For functional annotation, m6A peaks were assigned to nearby genes based on genomic proximity, as previously described [37].

### Immune cell marker gene analysis

Immune cell lineage-specific marker genes were selected based on established human immunophenotyping standards: B cells (CD19, MS4A1, CD79A, CD79B); plasma cells (CD38, SDC1, PRDM1, XBP1); CD4 + T cells (CD3D, CD3E, CD4, IL7R); CD8 + T cells (CD8A, CD8B, GZMA, GZMB, PRF1); NK cells (NCAM1, NKG7, KLRD1, FCGR3A); monocytes (CD14, FCGR3A, CCR2, ITGAM); macrophages (CD68, CD163, MSR1); dendritic cells (ITGAX, CD1C, CD83, HLA-DRA); neutrophils (CSF3R, S100A8, S100A9, S100A12); platelets (ITGA2B, PPBP, PF4); and endothelial cells (PECAM1, VWF, CDH5). The cross-species validity of these markers in rhesus macaques is supported by comparative immunology studies demonstrating high conservation of immune-related genes between rhesus macaques and humans [38–40]. For each marker, log₂ fold change values for mRNA expression and m6A modification were extracted at 7, 14, and 21 days post-infection and visualized as heatmaps.

## quantitative Reverse Transcription PCR (qRT-PCR)

Total RNA was extracted with TRIzol reagent; 1 µg of RNA was reverse-transcribed to cDNA using the PrimeScript™ FAST RT Reagent Kit (Takara, #RR092A). qRT-PCR was performed with TB Green Premix Ex Taq™ II (Takara, #RR820A) on a CFX96 System (Bio-Rad). Relative gene expression was calculated using the 2^-(ΔΔCt) method and normalized to GAPDH. Primer sequences are provided in Table S1. qRT-PCR was performed to verify the transcriptional levels of m6A modification system-related genes (METTL3, WTAP, FTO, ALKBH5, YTHDF1-3) in rhesus macaque whole blood at 0, 7, 14, and 21 days post-infection, as well as in multiple human cell lines (HUVEC, THP-1, A549, Huh7, CaCo2) before and after MPXV infection; And quantify the expression of MPXV F3L in sh-C and sh-METTL3/YTHDF2 Huh7 cells (generated via lentiviral transduction followed by puromycin selection).

### m6A RNA immunoprecipitation followed by quantitative PCR (MeRIP‑qPCR)

MeRIP-qPCR was performed using the EpiQuik™ CUT&RUN m6A RNA Enrichment Kit (EpigenTek) following the manufacturer’s protocol. Briefly, fragmented RNA was immunoprecipitated with an anti-m6A antibody, and the immunoprecipitated (IP) and input RNAs were reverse-transcribed and analyzed by qPCR using primers listed in Table S1. Relative m6A levels were calculated as the percentage of input (% input = 2^[(Ct_input - Ct_IP)] × 100%).

### Western blot (WB)

Cells were lysed in RIPA buffer, and protein concentration was measured using a BCA assay (CWBIO, #CW0014S). Proteins (25 µg per lane) were separated by 10% SDS-PAGE, transferred to nitrocellulose membranes, blocked, and incubated overnight at 4 °C with primary antibodies, followed by incubation with IRDye-labeled secondary antibodies. Protein bands were visualized using a ChemiDoc XRS+ imager (Bio-Rad). Antibodies used in this study are listed in Table S2.

### Global m6A quantification by ELISA

Global m6A levels in whole blood RNA samples were assessed using two complementary approaches. Colorimetric quantification was performed with the EpiQuik™ m6A RNA Methylation Quantification Kit (Epigentek, #P-9005-48) following the manufacturer’s protocol. Briefly, 200 ng of total RNA per sample was bound to strip wells, followed by sequential incubation with capture and detection antibodies. Absorbance was measured at 450 nm, and relative m6A abundance was calculated based on a standard curve.

### Statistical analysis

R software (versions 4.4.2 and 4.5.1) was used for data processing and visualization, with ggplot2 (v3.3.5) [41] used to generate figures. Statistical analyses were performed using GraphPad Prism (v10.1.2). Comparisons between two groups were evaluated using Student’s t-test, while comparisons among multiple groups were assessed using one-way or two-way ANOVA. A P-value < 0.05 was considered statistically significant.

## Results

### Sustained transcriptomic reprogramming with coordinated immunometabolic shifts

**Fig. 1:**
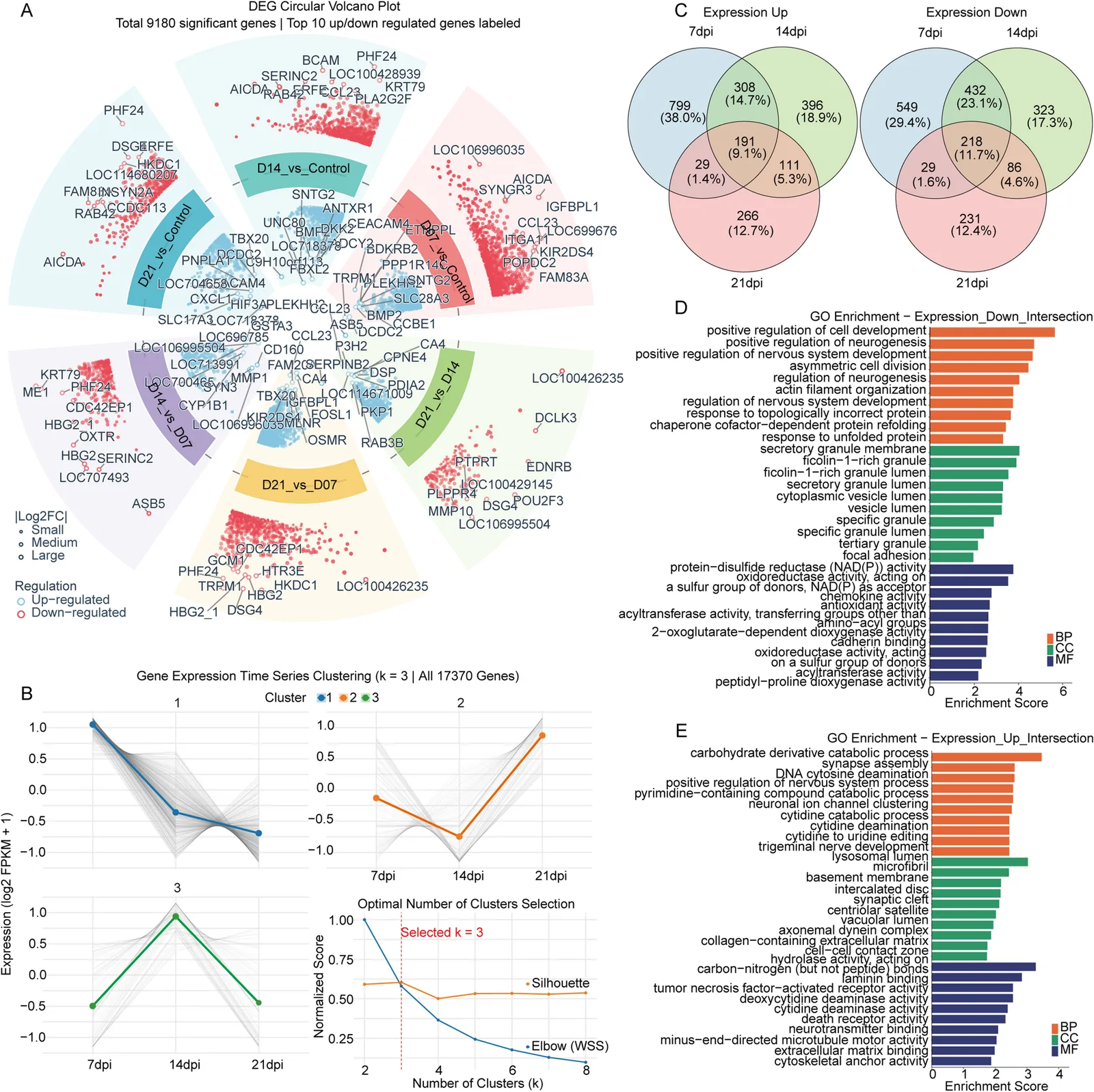
Dynamic transcriptomic profiling of MPXV infection in whole blood. **A **Volcano plots of differentially expressed genes (DEGs) at 7, 14, and 21 days post-infection (dpi) compared to uninfected controls (*P* < 0.05, |log₂FC| ≥ 1), with the top 10 most significant DEGs labeled. **B** Temporal expression clustering analysis. Top panel: three distinct expression clusters (k = 3) across 7, 14, and 21 dpi. Lines represent mean expression; shaded areas represent 95% confidence intervals. Bottom panel: combined plot of within‑cluster sum of squares (WSS, blue) and average silhouette width (orange) for k = 1–10, with the optimal k = 3 indicated by the dashed line. **C** Venn diagram showing the overlap of DEGs across all three time points, identifying 191 consistently upregulated and 218 consistently downregulated genes.** D-E** Bar plot of Gene Ontology (GO) enrichment for the consistently upregulated (D) and downregulated (E) core genes, showing biological process (BP), molecular function (MF), and cellular component (CC) terms

Viral load in whole blood, quantified by RNA detection, peaked at 7 days post-infection (dpi) and gradually declined through 21 dpi (Figure S1A). To characterize the host transcriptional response to dynamic viral replication, we performed RNA-seq on whole blood samples at 7, 14, and 21 dpi. Differential expression analysis revealed distinct gene sets at each time point (Fig. 1A). Among the most significantly altered genes, CCL23 was prominently upregulated at both early time points (7 and 14 dpi), implicating it in the initial host response, while AICDA emerged specifically at later stages (14 and 21 dpi), suggesting its role in adaptive immune mechanisms. qPCR validation confirmed the time‑specific upregulation of CCL23 and AICDA, and additionally revealed significant alterations in HBG2 (Figure S1B). Temporal clustering analysis grouped all expressed genes into three distinct expression trajectories (Fig. 1B; average silhouette width = 0.6068). Cluster 1 exhibited sustained downregulation over time. Cluster 2 decreased at 14 dpi but recovered by 21 dpi. Cluster 3 displayed transient upregulation peaking at 14 dpi. These patterns suggest dynamic host response programs with phased transcriptional regulation.

We next identified a persistent transcriptional signature consisting of genes consistently altered across all three time points, yielding 191 upregulated and 218 downregulated genes (Fig. 1C, Figure S1-1A). Heatmap visualization confirmed these distinct expression patterns (Figure S1-1B , Figure S1C). Functional enrichment and GSEA showed that downregulated genes were associated with immune pathways (e.g., endocytosis, chemokine signaling; Figure S1-1C , Figure S1E), whereas upregulated genes were linked to metabolic pathways, particularly glucose metabolism (Figure S1D, S1F). GO analysis indicated that downregulated genes were involved in cytoskeleton organization, while upregulated genes were enriched in base metabolism and neurotransmitter binding (Fig. 1D-E). The findings illustrate sustained transcriptomic reprogramming characterized by metabolic upregulation and concurrent downregulation of immune signaling.

### Dynamic m6A epitranscriptomic remodeling upon MPXV infection

**Fig. 2:**
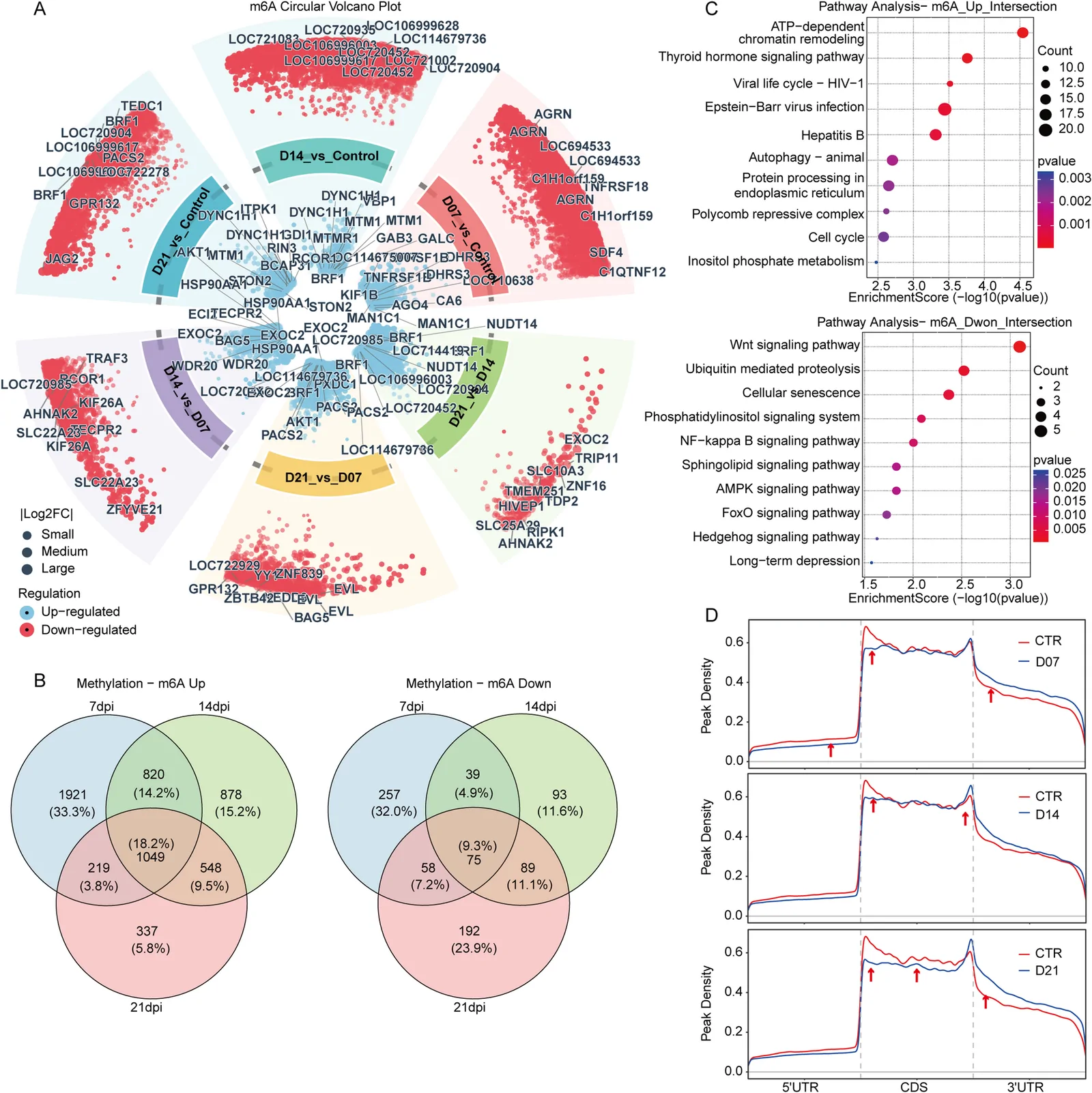
Dynamic m6A methylome alterations in MPXV-infected macaques. **A** Circular volcano plots of differentially m6A‑modified genes (DMGs) at 7, 14, and 21 dpi compared to uninfected controls (*P* < 0.05, |log₂FC| ≥ 2). **B** Venn diagram showing the overlap of DMGs across all three time points, identifying 1,049 consistently hypermethylated and 75 consistently hypomethylated sites. **C** Bubble plot of KEGG pathway enrichment for hypermethylated and hypomethylated DMGs. **D** Density distribution of differential m6A peaks across genomic regions (5′UTR, CDS, 3′UTR) at 7, 14, and 21 dpi. Red arrows indicate significant alterations

To profile host RNA methylation changes upon MPXV infection, we performed MeRIP-seq. This revealed a global increase in m6A levels during infection (Figure S2A), which was validated by ELISA on the same whole blood RNA samples (Figure S2B). Spearman correlation analysis further revealed a positive trend between global m6A levels and viral load, although this did not reach statistical significance (ρ = 0.225, *p* = 0.481; Figure S6A). We next identified DMGs at each time point (top genes annotated in Fig. 2A). Among these, AGRN exhibited significant m6A changes at 7 dpi, validated by m6A‑MeRIP‑qPCR (Figure S2C). Intersection analysis of m6A peaks across all three time points identified 1,049 consistently hypermethylated sites and 75 hypomethylated sites (Fig. 2B, Figure S2-1A). Functional enrichment revealed that hypermethylated genes were enriched in chromatin remodeling and autophagy, whereas hypomethylated genes were enriched in Wnt signaling and ubiquitin‑mediated proteolysis (Fig. 2C, Figure S2-1B).

Genomic distribution analysis revealed dynamic changes in m6A peak locations. At 7 and 14 dpi, peaks decreased in 5′UTR and start codon regions, while increasing in 3′UTR and CDS regions. By 21 dpi, modifications within CDS regions decreased, whereas those in 3′UTR and stop codon‑proximal regions increased (Fig. 2D, Figure S2D). Motif analysis confirmed enrichment of the canonical GGACU motif (Figure S2E). These results indicate that MPXV infection is associated with dynamic remodeling of the host m6A methylome, characterized by time‑dependent, pathway‑specific, and region‑biased alterations, which may influence post‑transcriptional regulation of host immune and metabolic responses.

### Coordinated reprogramming of the m6A epitranscriptomic machinery during MPXV infection

**Fig. 3:**
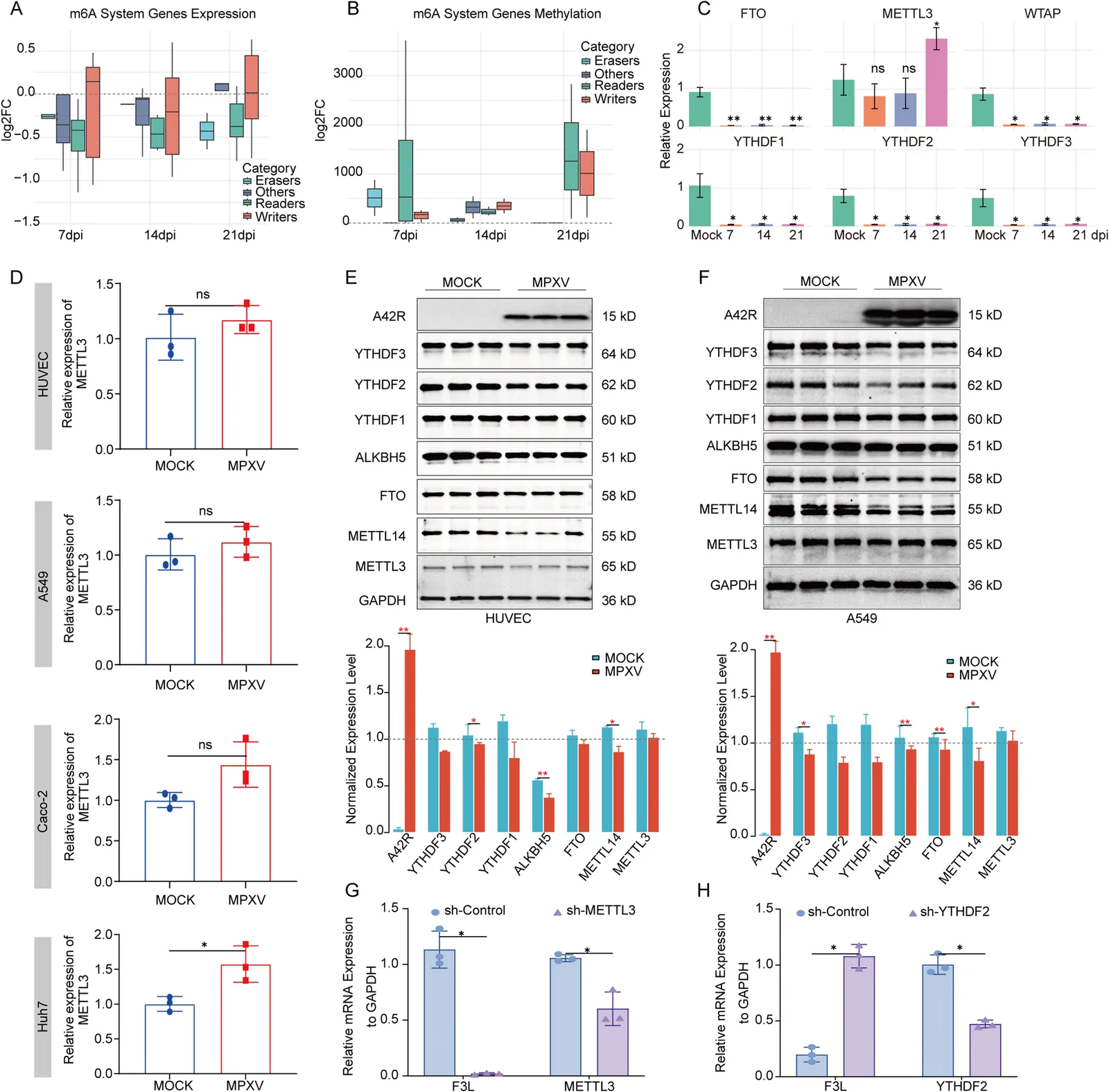
Coordinated reprogramming of the m6A epitranscriptomic machinery during MPXV infection. **A** Transcriptional changes (log₂FC) of key m6A regulatory components (writers, readers, erasers) at 7, 14, and 21 dpi based on RNA‑seq data. **B** Corresponding m6A modification changes (log₂FC) of these regulatory component transcripts based on MeRIP‑seq data. **C** qPCR validation of selected m6A regulators (FTO, WTAP, YTHDF1‑3, METTL3) in whole blood RNA samples at 7, 14, and 21 dpi. **D** qPCR analysis of METTL3 transcript levels in four human cell lines (HUVEC, A549, Huh7, CaCo2) at 48 h post‑infection (MOI = 0.1). **E-F** Western blot analysis of METTL3, YTHDF1‑3, FTO, and ALKBH5 protein levels in MPXV‑infected HUVEC (E) and A549 (F) cells. **G-H** Effects of METTL3 (G) or YTHDF2 (H) knockdown on MPXV replication in Huh7 cells. Viral replication was assessed by qPCR for viral RNA

To investigate the basis for the global m6A increase, we examined the expression and m6A modification of key m6A regulatory components, including writers, readers, and erasers. Transcriptomic and epitranscriptomic profiling revealed divergent patterns among these components (Fig. 3A-B, Figure S3A-B). METTL3 showed both increased transcription and elevated m6A modification. In contrast, most readers (e.g., YTHDF1-3) and erasers (e.g., ALKBH5) were transcriptionally downregulated, whereas their own transcripts exhibited increased m6A modification. qPCR using whole blood RNA from infected monkeys at 7, 14, and 21 dpi confirmed the downregulation of FTO and YTHDF1-3, as well as a late upregulation of METTL3 (Fig. 3C).

We next assessed the consistency of these changes across different cellular contexts. In four human cell lines (HUVEC, A549, Huh7, CaCo2) following MPXV infection, qPCR confirmed METTL3 upregulation across all cell lines (Fig. 3D). ALKBH5 showed downregulation in a subset of cell lines at the transcriptional level, while YTHDF family members exhibited no significant changes (Figure S3C). Spearman correlation analysis revealed that among the m6A regulatory genes, METTL3 showed positive trends with viral load (ρ = 0.423), whereas YTHDF3 showed a negative trend (ρ = -0.373), though these did not reach statistical significance after FDR correction (Figure S6B-H).Western blotting revealed no significant change in METTL3 protein levels. In contrast, protein levels of YTHDF1-3, FTO, and ALKBH5 were downregulated upon infection (Fig. 3E-F, Figure S3D). This pattern—unchanged methyltransferase protein levels coupled with markedly reduced demethylase and reader protein levels suggests that decreased m6A turnover, rather than increased methylation deposition, likely underlies the global m6A increase.

Analysis of public datasets GSE219036 [42] and GSE307689 [43] were consistent with these findings. In colon and skin tissues, MPXV infection showed upregulation of methyltransferases and downregulation of demethylases across both Congo and West African strains (Figure S2F). Similarly, in neural cell models, methyltransferase expression increased while demethylases were downregulated (Figure S2G). To preliminarily assess functional relevance, we knocked down METTL3 or YTHDF2 in Huh7 cells, which resulted in reduced viral replication (Fig. 3G-H). Together, these findings suggest a link between reprogramming of m6A regulatory components and the epitranscriptomic remodeling observed during MPXV infection.

### Integrated transcriptome and m6A methylome analysis reveals co‑regulated targets in MPXV infection

**Fig. 4:**
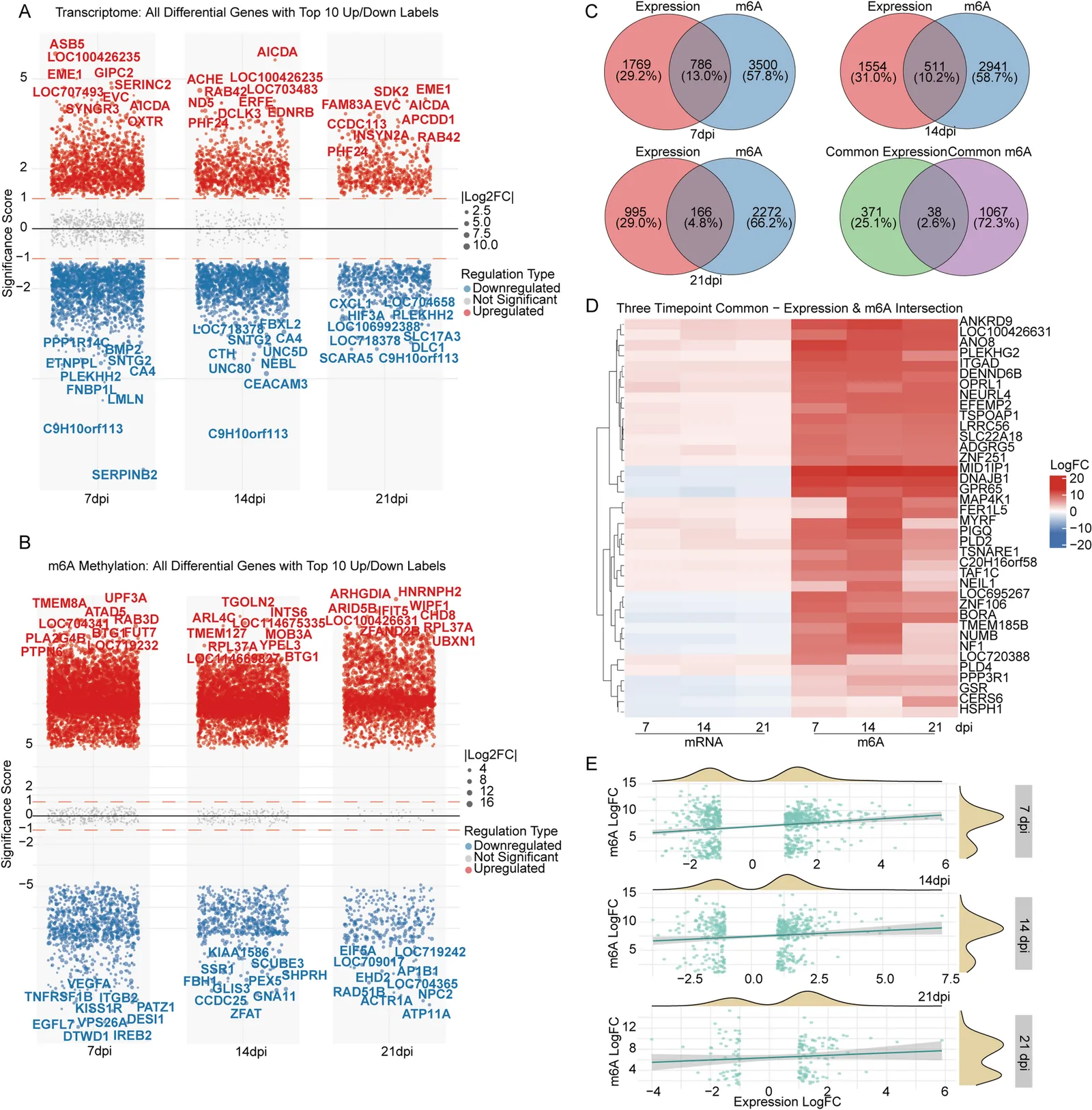
Integrated transcriptome and m6A methylome analysis reveals co‑regulated targets. **A** Columnar volcano plots of DEGs at each time point (column height: significance; bubble size: Log_2_FC; color: regulation direction). **B** Columnar volcano plots of differentially m6A-modified genes (column height: significance; bubble size: Log_2_FC; color: regulation direction). **C** Venn diagram of overlapping DEGs and differentially m6A-modified genes, identifying 38 core genes across infection. **D** Parallel heatmaps of expression and m6A modification levels for 38 core genes. **E** Correlation scatter plots of m6A modification vs. gene expression changes at each time point

Following the separate transcriptome and m6A methylome analyses, we performed an integrated study to identify genes co‑regulated at both the transcriptional and epitranscriptomic levels during MPXV infection. Column‑type volcano plots display DEGs and DMGs at each time point, with the ten most significant genes labeled (Fig. 4A‑B). Notably, the top‑ranking DEGs and DMGs showed minimal overlap, indicating that m6A‑mediated regulation is largely gene‑specific rather than genome‑wide. Venn analysis identified intersection genes exhibiting concurrent changes in both mRNA expression and m6A modification: 786 at 7 dpi, 511 at 14 dpi, and 166 at 21 dpi (Fig. 4C). Cross‑time‑point comparison identified 38 intersection genes consistently altered across all three stages, representing candidate genes with dual‑layer regulation. Correlation analysis with viral load revealed that several of these 38 genes showed positive trends, with MYRF (ρ = 0.817, *p* = 0.001) and SLC22A18 (ρ = 0.782, *p* = 0.003) exhibiting the strongest associations (Figure S8-9).

Functional annotation of the 786 intersection genes at 7 dpi revealed KEGG enrichment in pathways such as efferocytosis, DNA replication, and cytokine‑cytokine receptor interaction (Figure S2-1C). GO analysis highlighted terms related to regulation of phosphorylation, transferase activity, and cullin‑RING ubiquitin ligase complexes (Figure S4A), suggesting early activation of signaling cascades and protein turnover processes. A heatmap of the 38 consistently altered intersection genes showed that approximately one‑third exhibited an inverse correlation between expression level and m6A modification status (Fig. 4D). Correlation analysis across all intersection genes revealed both positive and negative relationships (Fig. 4E, Figure S4B). Time point‑specific quadrant plots further illustrated these expression‑modification relationships (Figure S4C). For example, PIM3 (quadrant IV) and INSYN2A (quadrant I) displayed distinct regulatory patterns. Time‑series analysis of selected intersection genes, including DNAJB1, TBKBP1, and ZNF177, illustrated the dynamic trajectories of both transcriptional and m6A modification levels across infection stages (Figure S4D).

### Genomic context influences the regulatory outcome of m6A modifications

**Fig. 5:**
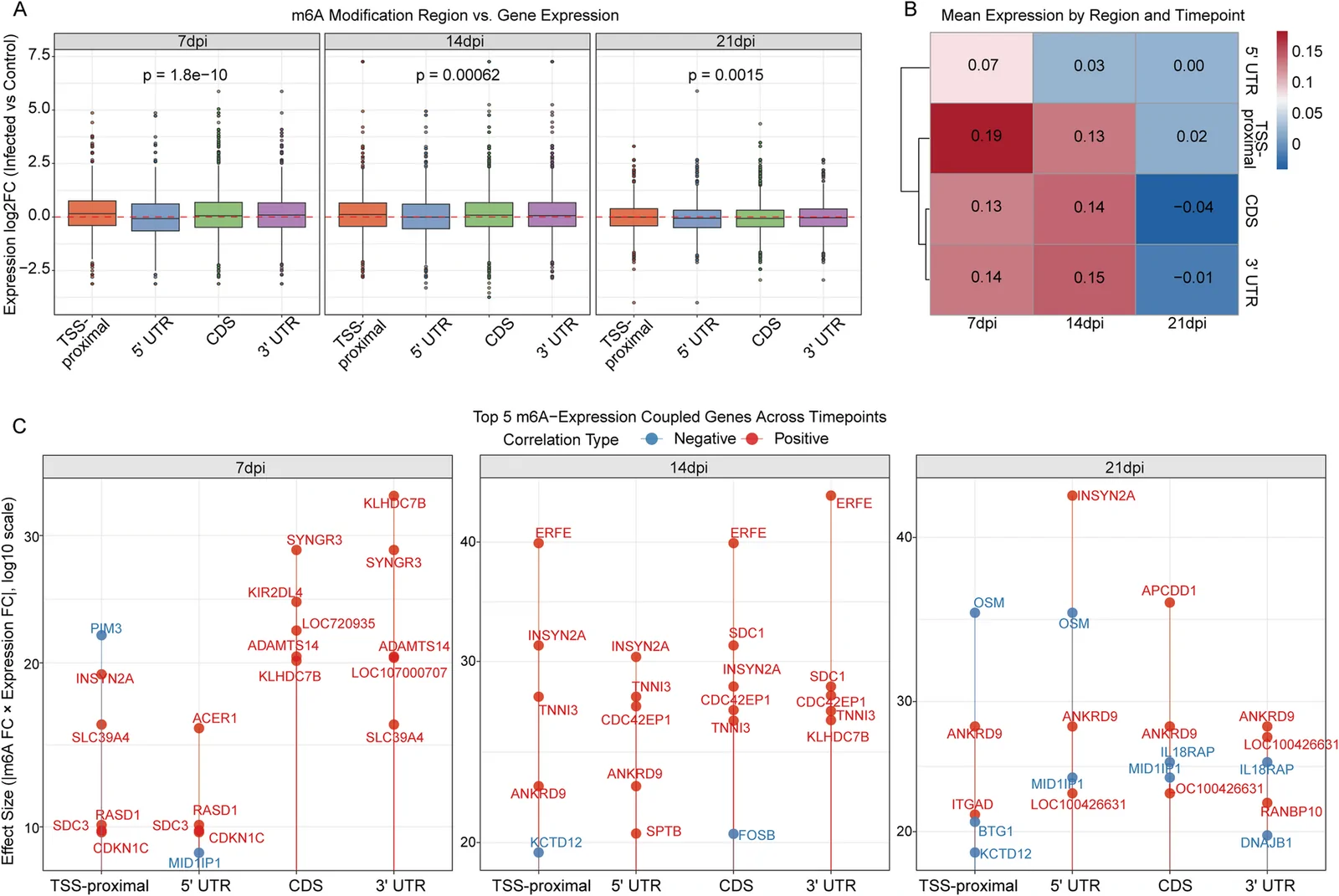
Genomic context influences the regulatory outcome of m6A modifications. **A** Box plots showing the distribution of m6A modification levels across different genomic regions (TSS‑proximal, 5′UTR, CDS, 3′UTR) and their association with gene expression changes (log₂FC) at 7, 14, and 21 dpi. **B** Heatmap of mean expression levels for genes with m6A modifications in each genomic region across 7, 14, and 21 dpi. **C** Top five m6A‑expression coupled genes across three time points based on effect size (|m6A FC × Expression FC|, log₁₀ scale)

To determine whether the regulatory effect of m6A modifications depends on their genomic position, we analyzed location‑specific m6A patterns within the previously identified intersection genes. Boxplot analysis revealed distinct associations between m6A modification levels in different genomic regions and the magnitude of gene expression changes (log₂FC). At 7 and 14 dpi, m6A modifications located in regions proximal to transcription start sites (TSS‑proximal) and within the 5′UTR showed a significant positive correlation with gene expression changes, with the TSS‑proximal region exhibiting the strongest effect at 7 dpi. By 21 dpi, this positive correlation became weak and non‑significant. In contrast, m6A modifications within the CDS and 3′UTR regions showed a weak negative correlation with expression changes across all time points (Fig. 5A). A complementary heatmap of mean expression levels across time points further illustrated these temporal dynamics (Fig. 5B).

We further identified the top five m6A‑expression coupled genes across the three time points based on effect size (|m6A FC × Expression FC|, log₁₀ scale), including ERFE, INSYN2A, KLHDC7B, SDC1, and SYNGR3 (Fig. 5C). Among them, ERFE and INSYN2A showed consistently high coupling scores across all time points, suggesting sustained dual‑layer regulation. Several representative genes exhibited strong correlations between expression and m6A modification in specific genomic regions, including PIM3 and INSYN2A. qPCR analysis revealed early upregulation of PIM3 and pronounced late upregulation of INSYN2A(Figure S1A). However, m6A‑MeRIP‑qPCR revealed no significant change in m6A modification of PIM3 (Figure S2B). Overall, these analyses suggest that the genomic context of m6A modifications may influence regulatory outcomes, particularly for TSS‑proximal and 5′UTR regions during early infection.

### Dynamic immune cell alterations revealed by cross‑omics profiling in peripheral blood

**Fig. 6:**
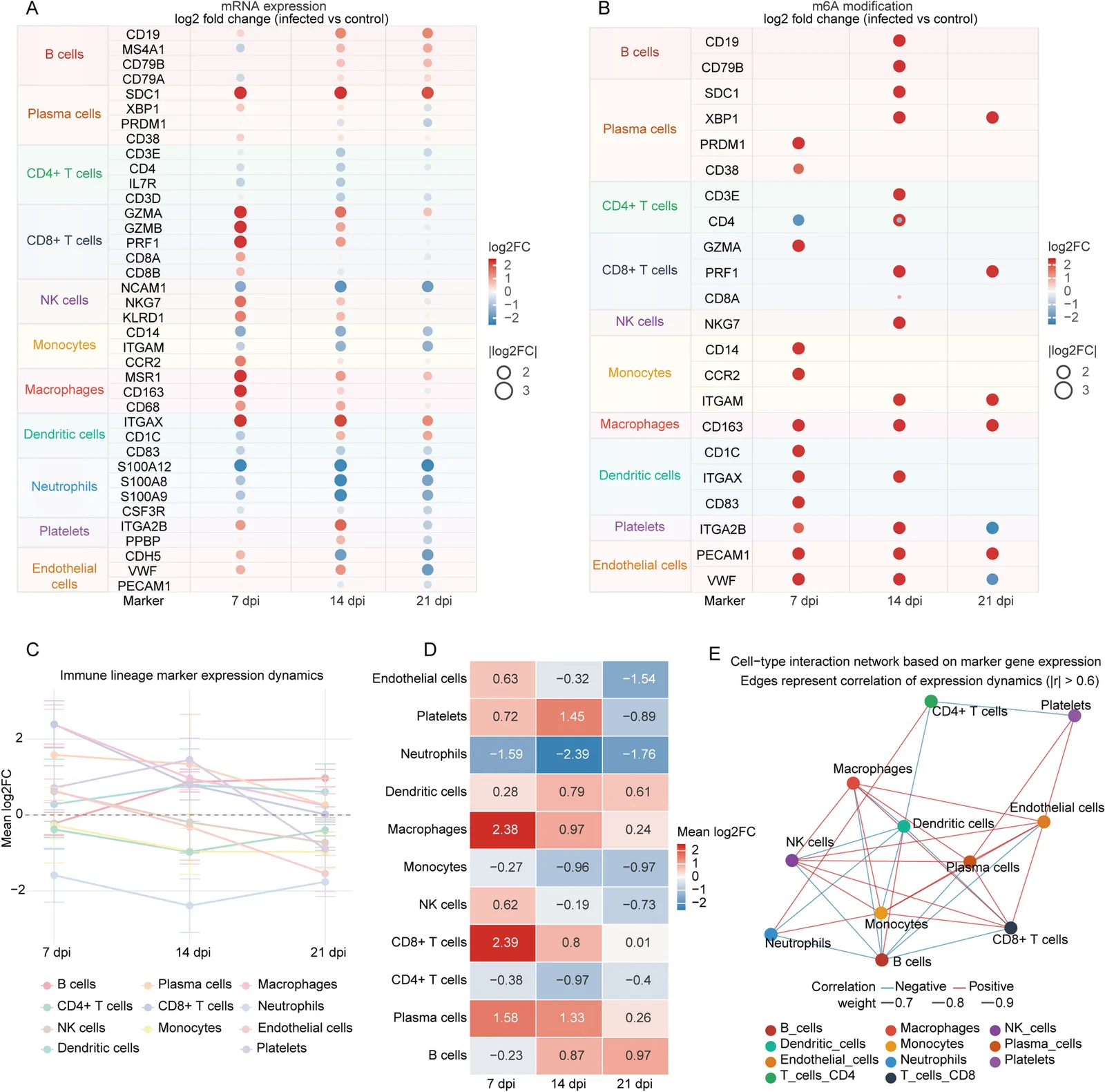
Dynamic immune cell alterations revealed by cross‑omics profiling. **A **Bubble plot showing expression changes (size: expression level; color: log₂FC) of immune cell lineage‑specific marker genes at 7, 14, and 21 dpi. **B** Bubble plot showing m6A modification changes (size: modification level; color: log₂FC) of immune cell lineage‑specific marker genes. **C **Line plot showing the temporal dynamics of lineage‑specific marker signature activities across 7, 14, and 21 dpi. **D** Heatmap visualizing the mean log₂FC of immune cell lineage‑specific marker genes at 7, 14, and 21 dpi. **E** Correlation network of immune cell signatures. Red edges indicate positive correlations; blue edges indicate negative correlations

To characterize the immune response in peripheral blood during MPXV infection, we analyzed the expression and m6A modification of immune cell lineage‑specific marker genes. This analysis revealed distinct regulatory patterns across immune subsets (Fig. 6A‑B). Markers for macrophages, NK cells, CD8 + T cells, platelets, and endothelial cells showed pronounced transcriptional upregulation at 7 dpi, followed by gradual downregulation at 14 and 21 dpi. In contrast, B cell markers exhibited progressively increased expression over the infection time course. m6A modification levels of most immune markers were elevated at 14 dpi. Notably, markers such as PRF1 (CD8 + T cells), ITGAM and CD163 (monocytes/macrophages), and PECAM1 (endothelial cells) showed sustained m6A enrichment across all three time points.

Based on these expression patterns, we examined the temporal dynamics of lineage‑specific marker signatures. The overall activity of CD8 + T cell, CD4 + T cell, NK cell, and monocyte/macrophage signatures gradually decreased over time, whereas the B cell signature showed a progressive increase. Neutrophil signature activity remained consistently low throughout infection (Fig. 6C‑D). We next examined coordinated changes in marker gene expression to infer potential inter‑cellular associations. Correlation analysis revealed positive correlations between monocyte/macrophage and CD8 + T cell signatures, as well as between monocyte/macrophage and both endothelial cell and NK cell signatures. Negative correlations were observed between dendritic cell signature and both CD4 + and CD8 + T cell signatures, as well as between dendritic cell and monocyte/macrophage signatures (Fig. 6E). These correlation patterns suggest potential coordinated responses between innate and adaptive immune compartments during MPXV infection.

### Cross-Omics Identifies Host m6A-Regulated Targets and Biomarkers in MPXV Infection

**Fig. 7:**
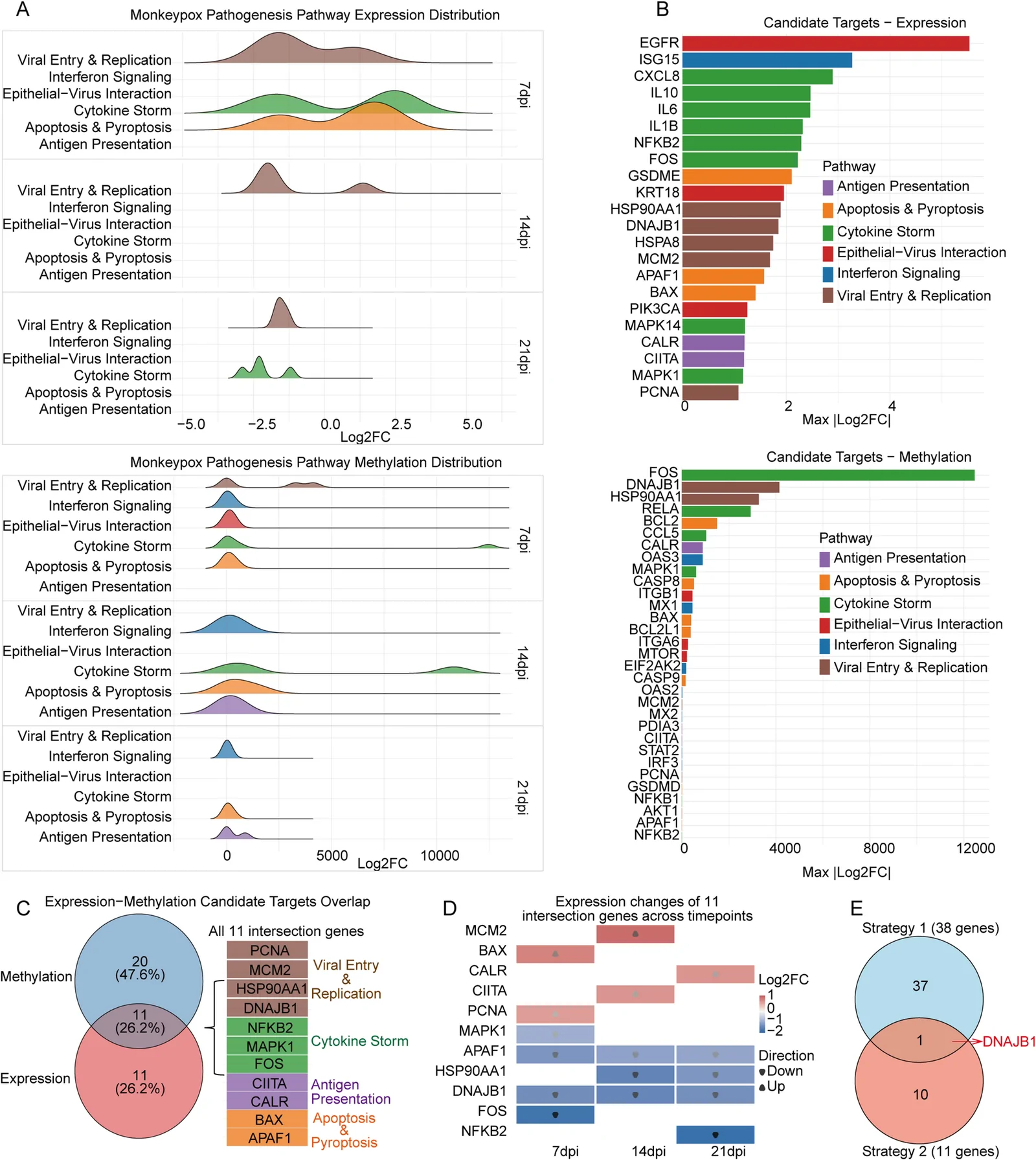
Cross-omics identification of m6A-regulated targets and biomarkers. **A** Density plots showing the distribution of log₂ fold change values for gene expression and m6A modification across six key biological processes including viral entry/replication, inflammatory signaling, apoptosis/pyroptosis, antigen presentation, interferon response, and virus–epithelium interaction (x-axis: log₂FC). **B **Bar plots of candidate genes with significant transcriptional/m6A alterations (x-axis: max|log₂FC|). **C** Venn diagram identifying 11 candidate co-regulated targets. **D** Heatmap of 11 targets’ expression dynamics during infection. **E** Venn diagram showing the overlap between the 11 genes identified by pathway-driven dual-layer analysis and the 38 core genes defined by concurrent expression and m6A changes at all three time points (7, 14, 21 dpi)

Focusing on key MPXV pathogenic processes reported in the literature [44, 45], integrated analysis identified pronounced dual‑layer perturbations in pathways for viral entry/replication, inflammatory signaling, and apoptosis/pyroptosis, each with distinct temporal dynamics (Fig. 7A, Figure S7A-B). From these pathways, we identified 22 genes with significant transcriptional changes and 31 genes with significant m6A modifications (Fig. 7B). Intersection analysis refined a set of 11 genes altered at both levels (Fig. 7C), including PCNA, MCM2, HSP90AA1, DNAJB1 (replication/stress); NFKB2, MAPK1, FOS (inflammation); CIITA, CALR (antigen presentation); and BAX, APAF1 (cell death). Their transcriptional trajectories throughout infection are shown in Fig. 7D. qPCR and m6A‑MeRIP‑qPCR analyses on whole‑blood RNA samples confirmed that expression and m6A modification patterns for most of these 11 genes were consistent with sequencing results across time points (Figure S1B, Figure S2C). Among these 11 genes, PCNA, BAX, and MCM2 showed significant positive correlations with viral load after FDR correction (ρ = 0.746, 0.746, and 0.725, respectively; all FDR < 0.05), suggesting their potential association with viral replication (Figure S10). We next intersected this 11-gene set with the 38 core genes identified earlier by concurrent expression and m6A changes at all three time points (7, 14, 21 dpi). DNAJB1 was the only gene shared between the two independent selection strategies (Fig. 7E), cross‑validating the biological relevance of both approaches.

Comparison with public datasets (GSE219036, GSE307689, GSE287262 [46]) revealed heterogeneity in host responses across tissues, experimental contexts, and viral strains (Figure S7C‑F). For example, BAX and MCM2 were upregulated in our peripheral blood model during peak viral replication, but were downregulated in external skin datasets from cases infected with the 2022 isolate and in lung datasets from infections with three different viral strains. Similarly, PCNA showed early upregulation in our study but was generally suppressed in various infected tissues and cell lines from public datasets. Given no detectable m6A on MPXV viral transcripts in our MeRIP‑seq data, these host‑centric events reflect virus‑induced endogenous pathway perturbations.

## Discussion

Our integrated multi-omics analysis reveals that MPXV infection is associated with reprogramming of the host m6A epitranscriptome, characterized by a global increase in m6A peak abundance. This modification occurs alongside a divergent transcriptional response that suppresses immune pathways while upregulating metabolic processes, a pattern consistent with a viral strategy of concurrent immune evasion and cellular resource exploitation [11, 47]. Mechanistically, this global m6A increase was associated with a non-canonical remodeling of the m6A regulatory machinery. While METTL3 transcript levels were upregulated, its protein levels showed no significant change. In contrast, the erasers FTO and ALKBH5, as well as the readers YTHDF1-3, were significantly downregulated at the protein level. This pattern suggests that the observed m6A hypermethylation is primarily consistent with reduced m6A removal and impaired reader‑mediated turnover, rather than with enhanced writing activity. Such a mechanism may allow the virus to achieve sustained epitranscriptomic reprogramming without constitutively activating the writer machinery. Knockdown of METTL3 or YTHDF2 impaired MPXV replication, further supporting the functional relevance of this regulatory circuit. Notably, we did not detect m6A modifications on MPXV transcripts in our MeRIP‑seq data. The absence of m6A signals on viral transcripts may be partially attributable to technical limitations of MeRIP‑seq, including antibody specificity and peak‑level resolution. Additionally, as a substantial proportion of viral RNA may be released into the extracellular space during infection, the intracellular viral RNA pool available for immunoprecipitation could be markedly reduced.

The functional impact of m6A remodeling was highly context dependent. m6A peaks in regions proximal to transcription start sites and within 5′UTRs predominantly correlated with gene upregulation, supporting m6A’s emerging role in transcriptional regulation via chromatin associated RNAs [12, 37], whereas coding region modifications showed weak negative correlations, explaining the limited global concordance between expression and m6A changes. Position specific patterns in genes such as PIM3 and INSYN2A provide mechanistic hypotheses, but base resolution mapping and reader protein profiling are needed for definitive insight [48]. Through integrative analysis, we prioritized 11 host genes under dual-layer regulation, including PCNA, MCM2, HSP90AA1, DNAJB1, NFKB2, MAPK1, FOS, CIITA, CALR, BAX, and APAF1. Their concerted upregulation across infection time points points to coordinated functions in DNA replication, inflammatory signaling, antigen presentation, and cell death [49–52]. Notably, DNAJB1 was the only gene shared between this pathway-driven set and the 38 core genes identified through independent time‑point intersection analysis. This cross‑strategy convergence suggests its potential biological relevance as a candidate host factor potentially co-opted by MPXV.

Analysis of immune lineage specific marker signatures revealed dynamic changes during infection. The activities of CD8 + T cell, CD4 + T cell, NK cell, and monocyte/macrophage signatures gradually decreased over time, whereas the B cell signature showed a progressive increase. Correlation analysis further suggested potential coordinated responses between innate and adaptive immune compartments. These findings provide a system level view of immune remodeling during MPXV infection. Future studies using single cell RNA sequencing could dissect cell type specific contributions with higher resolution [53].

Comparison with public datasets revealed heterogeneity in host responses. These discrepancies likely reflect differences in tissue context (peripheral blood versus local infection sites), viral strain (clade II versus other isolates), and temporal sampling (time series versus single time points). Importantly, core m6A regulatory trends, such as methyltransferase upregulation, remained consistent across datasets (Figure S3C‑D). Therefore, this heterogeneity does not invalidate our conclusions but rather highlights the context dependent nature of host responses. The 11 candidate host factors identified here should be interpreted within the context of peripheral blood responses to clade II MPXV infection, and their generalizability warrants further investigation.

Several limitations should be acknowledged. The sample size (*n* = 3 per group) is modest but comparable to previous non-human primate multi-omics studies, and we mitigated this with rigorous multiple testing corrections and orthogonal validations. Our analyses are correlative and do not establish causality. Functional validation was primarily limited to METTL3 and YTHDF2 in Huh7 cells, which may not fully recapitulate native physiological contexts. Additionally, as a limitation of MeRIP-seq, our study achieves only peak-level resolution, precluding precise assignment of m6A sites, and is subject to inherent antibody bias. Future studies using single-base-resolution methods (e.g., Nanopore direct RNA sequencing) and physiologically relevant models will be required to validate and extend our findings. Overall, this study provides a hypothesis-generating framework identifying m6A epitranscriptomic remodeling as a key correlate of MPXV infection and nominates DNAJB1 and the other 10 dual-regulated genes as candidates for further mechanistic investigation.

## Supplementary Information

Supplementary Material 1.

Supplementary Material 2.

Supplementary Material 3

## Data Availability

The raw sequencing data generated in this study have been deposited in the NCBI Gene Expression Omnibus (GEO) database under the SuperSeries accession number GSE326690 (NCBI tracking system #25716259). The data can be accessed via the following link: https://www.ncbi.nlm.nih.gov/geo/query/acc.cgi? acc=GSE326690.
