# Supplementary material for: Dynamic m6A epitranscriptomic remodeling coordinates host transcriptional reprogramming during MPXV infection
Source: Cell Commun Signal. 2026 Jul 18;24:549. doi: 10.1186/s12964-026-03090-w (PMC13659962; doi:10.1186/s12964-026-03090-w)
Supplement: Supplementary file 1 [file 12964_2026_3090_MOESM2_ESM.docx]

Table S1. Primers for validation

| Primers | Sequences |
| --- | --- |
| hPCNA-F | TTAGGCATTCTTAAAATTCA |
| hPCNA-R | GTACAAATTTGGTGACAGAA |
| hHSP90AA1-F | GTTTGGAGGAGCAAAGTTAA |
| hHSP90AA1-R | TGACTCGAGCACTACATTTC |
| hDNAJB1-F | GGTAATTTTTTGTTTCATGG |
| hDNAJB1-R | AAGACCCTTTTATCAAAAGG |
| hFOS-F | GAAAGCATCCATGTGTGGAC |
| hFOS-R | CTACCAGCTCTCTGAAGTGT |
| hBAX-F | TCAGGATGCGTCCACCAAGAAG |
| hBAX-R | TGTGTCCACGGCGGCAATCATC |
| hCALR-F | AGATTGACAACAGCCAGGTG |
| hCALR-R | GTCTTCCGGTTTTGAAGCAT |
| hCIITA-F | CTACTTCAGGCAGCAGAGGAGA |
| hCIITA-R | GCTGTGTCTTCCGAGGAACTTC |
| hMCM2-F | GTGTGTCTTACTTGGTTGCT |
| hMCM2-R | CACACGCAGGCATCCTGAAC |
| hAPAF1-F | GTGACATTTCTCACGATGCT |
| hAPAF1-R | CCTCAATTCATGAAGTGGCA |
| hINSYN2A-F | GTGACACATGCAGGAACAGTGC |
| hINSYN2A-R | GCGAAGGTATGACCTGAGTTTCC |
| hTBKBP1-F | AACGGCTCAACCAGTTCCAGCA |
| hTBKBP1-R | CCAAGTCACTCTTCTCCACGCA |
| hPIM3-F | CCTGCGGCCCTCAGAGCGGC |
| hPIM3-R | ACAGCCGCAGGTCACAGCTC |
| hNF1-F | GGCCAATGTGGTTCCTTGTT |
| hNF1-R | ACATGTTCCAGAGCAGTTGG |
| hMAP4K1-F | CAAGATCCAGGACACCAAAGGC |
| hMAP4K1-R | ACCACTGAAGCAGGACAACGGA |
| hRPAIN-F | CATCTGTCCTGTATGTACAA |
| hRPAIN-R | GTGTGGGGACAATGTGCACT |
| hMAPK1-F | ACACCAACCTCTCGTACATCGG |
| hMAPK1-R | TGGCAGTAGGTCTGGTGCTCAA |
| hCCL23-F | CCGTGTTCACTCCTGGAGAGTT |
| hCCL23-R | GCTTCAGCATTCTCACGCAAACC |
| hAICDA-F | TGAAGAGGCGTGACAGTGCTAC |
| hAICDA-R | GAGATGTAGCGGAGGAAGAGCA |
| hHBG2-F | GGAAGATGCTGGAGGAGAAACC |
| hHBG2-R | GTCAGCACCTTCTTGCCATGTG |
| hTRPM1-F | ACTACGTCATCCTGGTGCGGAT |
| hTRPM1-R | CTGGCTGAGTTTGCCTGGTTCT |
| hAGRN-F | CCGTCCCCGAAACTCTGGAT |
| hAGRN-R | AACCAGCGGTTGGTGTTGA |
| hMETTL3-F | CTATCTCCTGGCACTCGCAAGA |
| hMETTL3-R | GCTTGAACCGTGCAACCACATC |
| hWTAP-F | GCAACAACAGCAGGAGTCTGCA |
| hWTAP-R | CTGCTGGACTTGCTTGAGGTAC |
| hFTO-F | CCAGAACCTGAGGAGAGAATGG |
| hFTO-R | CGATGTCTGTGAGGTCAAACGG |
| hALKBH5-F | CCAGCTATGCTTCAGATCGCCT |
| hALKBH5-R | GGTTCTCTTCCTTGTCCATCTCC |
| hYTHDF1-F | CAAGCACACAACCTCCATCTTCG |
| hYTHDF1-R | GTAAGAAACTGGTTCGCCCTCAT |
| hYTHDF2-F | TAGCCAGCTACAAGCACACCAC |
| hYTHDF2-R | CAACCGTTGCTGCAGTCTGTGT |
| hYTHDF3-F | GCTACTTTCAAGCATACCACCTC |
| hYTHDF3-R | ACAGGACATCTTCATACGGTTATTG |
| vF3L-F | CTCATTGATTTTTCGCGGGATA |
| vF3L-R | GACGATACTCCTCCTCGTTGGT |
| F3L-Probe | FAM-CATCAGAATCTGTAGGCCGT-MGB |
| mGAPDH-F | AGCCCCATCACCATCTTCC |
| mGAPDH-R | AATGAGCCCCAGCCTTCTC |
| mHPRT1-F | ATGGAAGCCACAGGTAGTGC |
| mHPRT1-R | TCATCACTAATCGCCGTCCG |

Table S2. Antibodies for validation

| Antibody | Catalog No. |
| --- | --- |
| WTAP Monoclonal antibody | 60188-1-Ig |
| METTL14 Polyclonal antibody | 26158-1-AP |
| recombinant Anti-METTL3 antibody[EPR18810] | ab195352 |
| FTO Polyclonal antibody | 27226-1-AP |
| ALKBH5 Polyclonal antibody | 16837-1-AP |
| YTHDF1 Polyclonal antibody | 17479-1-AP |
| YTHDF2 Polyclonal antibody | 24744-1-AP |
| YTHDF3-specific Polyclonal antibody | 25537-1-AP |
| Anti-MPXV A42R/Profilin Polyclonal Antibody | PVV21901 |
| GAPDH Monoclonal antibody | 60004-1-Ig |
| GAPDH Polyclonal antibody | 10494-1-AP |

Table S3. Sequencing quality metrics for all samples

|  | Raw Reads | Clean Reads | Clean Ratio | Peak Number |
| --- | --- | --- | --- | --- |
| C-1.Input | 41,594,450 | 41,365,706 | 99.45% | - |
| C-2.Input | 40,936,294 | 40,740,664 | 99.52% | - |
| C-3.Input | 50,150,068 | 49,726,684 | 99.16% | - |
| 07-1.Input | 40,342,628 | 40,105,848 | 99.41% | - |
| 07-2.Input | 47,988,200 | 47,520,916 | 99.03% | - |
| 07-3.Input | 42,169,124 | 41,960,572 | 99.51% | - |
| 14-1.Input | 42,356,148 | 42,049,766 | 99.28% | - |
| 14-2.Input | 44,391,132 | 44,174,466 | 99.51% | - |
| 14-3.Input | 43,480,722 | 43,233,244 | 99.43% | - |
| 21-1.Input | 39,381,040 | 39,113,538 | 99.32% | - |
| 21-2.Input | 56,416,360 | 56,166,164 | 99.56% | - |
| 21-3.Input | 51,720,880 | 51,431,770 | 99.44% | - |
| C-1.IP | 46,102,754 | 45,505,030 | 98.70% | 15,075 |
| C-2.IP | 43,824,036 | 43,357,284 | 98.93% | 10,327 |
| C-3.IP | 46,649,842 | 46,178,618 | 98.99% | 9,767 |
| 07-1.IP | 42,831,274 | 42,324,128 | 98.82% | 49,945 |
| 07-2.IP | 39,482,756 | 39,036,318 | 98.87% | 24,489 |
| 07-3.IP | 49,975,670 | 49,493,234 | 99.03% | 25,398 |
| 14-1.IP | 46,171,472 | 45,652,448 | 98.88% | 24,175 |
| 14-2.IP | 49,470,748 | 48,985,362 | 99.02% | 39,478 |
| 14-3.IP | 51,572,972 | 50,956,188 | 98.80% | 43,749 |
| 21-1.IP | 40,986,966 | 40,421,330 | 98.62% | 37,702 |
| 21-2.IP | 38,858,086 | 38,371,596 | 98.75% | 12,182 |
| 21-3.IP | 42,687,646 | 42,229,004 | 98.93% | 16,993 |

Table S4. Spearman correlation between viral load and molecular features

| Gene | rho | p_value | n | p_adjusted |
| --- | --- | --- | --- | --- |
| PIM3 | 0.880303519004998 | 0.000157757375943142 | 12 | 0.00315514751886283 |
| AGRN | 0.767624668572358 | 0.0035576172578496 | 12 | 0.0352534638412797 |
| PCNA | 0.746497384116239 | 0.00528801957619195 | 12 | 0.0352534638412797 |
| TBKBP1 | 0.485927542490759 | 0.109227184929028 | 12 | 0.427939879960004 |
| RBM15 | 0.471842686186679 | 0.121455142891161 | 12 | 0.427939879960004 |
| RBM15B | 0.457757829882599 | 0.134548594648049 | 12 | 0.427939879960004 |
| METTL3 | 0.422545689122399 | 0.171175951984002 | 12 | 0.427939879960004 |
| ALKBH5 | 0.422545689122399 | 0.171175951984002 | 12 | 0.427939879960004 |
| YTHDF1 | 0.338036551297919 | 0.282516852729084 | 12 | 0.565033705458167 |
| YTHDF2 | 0.2464849853214 | 0.439943113216354 | 12 | 0.641716550595502 |
| INSYN2A | 0.22535770086528 | 0.481287412946627 | 12 | 0.641716550595502 |
| FTO | 0.19014556010508 | 0.553900040754205 | 12 | 0.649045033475684 |
| METTL14 | 0.176060704 | 0.584140530128115 | 12 | 0.649045033475684 |
| HSP90AA1 | -0.070424282 | 0.827831199192635 | 12 | 0.827831199192635 |
| WTAP | -0.133806135 | 0.678446686329941 | 12 | 0.714154406663095 |
| YTHDC2 | -0.176060704 | 0.584140530128115 | 12 | 0.649045033475684 |
| DNAJB1 | -0.225357701 | 0.481287412946627 | 12 | 0.641716550595502 |
| YTHDC1 | -0.239442557 | 0.453529963414165 | 12 | 0.641716550595502 |
| NF1 | -0.309866839 | 0.327005010215011 | 12 | 0.594554564027293 |
| YTHDF3 | -0.373248692 | 0.23206767137486 | 12 | 0.515705936388578 |
| MYRF | 0.816921665636638 | 0.00118060561747551 | 12 | 0.0448630134640694 |
| SLC22A18 | 0.781709524876439 | 0.00267006598302026 | 12 | 0.050731253677385 |
| PIGQ | 0.732412527812159 | 0.0067516524803181 | 12 | 0.0739177717125264 |
| LRRC56 | 0.718327671508079 | 0.00850035683818146 | 12 | 0.0739177717125264 |
| ANKRD9 | 0.690157958899919 | 0.0129864256750896 | 12 | 0.0739177717125264 |
| ANO8 | 0.690157958899919 | 0.0129864256750896 | 12 | 0.0739177717125264 |
| ZNF251 | 0.676073102595839 | 0.0157927720786675 | 12 | 0.0739177717125264 |
| OPRL1 | 0.661988246291759 | 0.0190217268823851 | 12 | 0.0739177717125264 |
| EFEMP2 | 0.661988246291759 | 0.0190217268823851 | 12 | 0.0739177717125264 |
| FER1L5 | 0.654945818139719 | 0.0208058566785112 | 12 | 0.0739177717125264 |
| LOC720388 | 0.647903389987679 | 0.0227090706649032 | 12 | 0.0739177717125264 |
| MAP4K1 | 0.633818533683599 | 0.0268907007358238 | 12 | 0.0739177717125264 |
| C20H16orf58 | 0.633818533683599 | 0.0268907007358238 | 12 | 0.0739177717125264 |
| ITGAD | 0.626776105531559 | 0.0291780677812604 | 12 | 0.0739177717125264 |
| TSPOAP1 | 0.626776105531559 | 0.0291780677812604 | 12 | 0.0739177717125264 |
| DENND6B | 0.605648821075439 | 0.036879648297599 | 12 | 0.0824368609005155 |
| PLD4 | 0.591563964771359 | 0.0427573315040295 | 12 | 0.0891545023138719 |
| PLEKHG2 | 0.577479108467279 | 0.0492695933839818 | 12 | 0.0891545023138719 |
| NEURL4 | 0.577479108467279 | 0.0492695933839818 | 12 | 0.0891545023138719 |
| TAF1C | 0.577479108467279 | 0.0492695933839818 | 12 | 0.0891545023138719 |
| ADGRG5 | 0.570436680315239 | 0.052774096020423 | 12 | 0.0911552567625488 |
| TSNARE1 | 0.556351824011159 | 0.0603000255410738 | 12 | 0.0916560388224322 |
| NEIL1 | 0.556351824011159 | 0.0603000255410738 | 12 | 0.0916560388224322 |
| PLD2 | 0.528182111402999 | 0.077527200453535 | 12 | 0.113308985278243 |
| LOC100426631 | 0.443672973578519 | 0.148524844747907 | 12 | 0.201569432157874 |
| LOC695267 | 0.380291120210159 | 0.222673422334491 | 12 | 0.282053001623688 |
| TMEM185B | 0.295781982385679 | 0.350596359398154 | 12 | 0.403717019913026 |
| GSR | 0.0352121407601999 | 0.913488219830278 | 12 | 0.913488219830278 |
| MID1IP1 | -0.140848563 | 0.662386795610918 | 12 | 0.68028914143824 |
| ZNF106 | -0.19014556 | 0.553900040754205 | 12 | 0.584672265240549 |
| DNAJB1 | -0.225357701 | 0.481287412946627 | 12 | 0.522540619770623 |
| NUMB | -0.225357701 | 0.481287412946627 | 12 | 0.522540619770623 |
| NF1 | -0.309866839 | 0.327005010215011 | 12 | 0.388318449630325 |
| HSPH1 | -0.373248692 | 0.23206767137486 | 12 | 0.284470048782086 |
| GPR65 | -0.408460833 | 0.187417731783862 | 12 | 0.245581855440923 |
| CERS6 | -0.478885114 | 0.115234156162271 | 12 | 0.162181404969122 |
| BORA | -0.563394252 | 0.0564495878391508 | 12 | 0.0916560388224322 |
| PPP3R1 | -0.612691249 | 0.0341681385534261 | 12 | 0.0811493290643869 |
| PCNA | 0.746497384116239 | 0.00528801957619195 | 12 | 0.0278238643352684 |
| BAX | 0.746497384116239 | 0.00528801957619195 | 12 | 0.0278238643352684 |
| MCM2 | 0.725370099660119 | 0.00758832663689138 | 12 | 0.0278238643352684 |
| CALR | 0.697200387051959 | 0.0117306361137115 | 12 | 0.0322592493127067 |
| CIITA | 0.422545689122399 | 0.171175951984002 | 12 | 0.376587094364804 |
| NFKB2 | 0.243389650224709 | 0.445890542859692 | 12 | 0.661770192801612 |
| APAF1 | 0.11972127858468 | 0.710931859519094 | 12 | 0.868916717190004 |
| FOS | -0.035212141 | 0.913488219830278 | 12 | 0.913488219830278 |
| HSP90AA1 | -0.070424282 | 0.827831199192635 | 12 | 0.910614319111898 |
| DNAJB1 | -0.225357701 | 0.481287412946627 | 12 | 0.661770192801612 |
| MAPK1 | -0.309866839 | 0.327005010215011 | 12 | 0.599509185394187 |

**Supplementary Figures Legend**

**Figure S1. Sustained transcriptomic reprogramming with coordinated immunometabolic shifts.** (A) Viral load in whole blood RNA samples at 7, 14, and 21 days post-infection (dpi) quantified by RNA detection. (B) qPCR validation of time‑specific expression patterns for CCL23, AICDA, and HBG2 in whole blood RNA samples at 7, 14, and 21 dpi. (C) Heatmap of normalized expression values for the top 50 upregulated genes from the 191 persistently upregulated core gene set. (D) Bar plot of KEGG pathway enrichment for the persistently upregulated core genes. (E) Representative GSEA plots for significantly downregulated pathways (e.g., endocytosis, chemokine signaling). (F) Representative GSEA plots for significantly upregulated pathways (e.g., glucose metabolism).

**Figure S2. Dynamic m6A epitranscriptomic remodeling upon MPXV infection.** (A) Statistical summary of overall m6A modification changes across infection time points. Bar plot showing the quantitative distribution of upregulated and downregulated modification peaks. (B) Validation of global m6A levels by ELISA assays on the same whole blood RNA samples. (C) MeRIP-qPCR validation of modification levels of significantly altered genes in whole blood RNA samples across different time points, presented as a heatmap. Rows represent genes, columns represent sample groups, and functional categories of genes are annotated. (D) Distribution changes of differential m6A modification peaks across genomic regions (5′UTR, CDS, start codon‑proximal (±100 bp), stop codon‑proximal (±100 bp) regions and 3′UTR) at 7, 14, and 21 dpi compared with uninfected controls. (E) Motif analysis of m6A peak sequences using HOMER, showing enrichment of the canonical GGACU motif. (F) Transcript expression levels (log₂(TPM+1)) of m6A machinery genes in (top) colon and (bottom) skin tissues from the GEO dataset GSE219036, following infection with Congo Basin or West African MPXV strains. (G) Expression levels of selected m6A methyltransferases and demethylases in MPXV-infected HMC3, SH-SY5Y, and U87MG cell lines from public GEO dataset GSE307689.

**Figure S3. Coordinated reprogramming of the m6A epitranscriptomic machinery.** (A) Transcript‑level changes (log₂FC) of m6A regulatory genes (writers, readers, erasers, and other related factors) at 7, 14, and 21 dpi. (B) Corresponding changes in self‑m6A modification levels (log₂FC) of the same m6A machinery transcripts. Bar colors indicate functional categories: writers (red), readers (green), erasers (blue), and others (purple). Positive and negative values indicate upregulation or downregulation, respectively, relative to uninfected controls. (C) qPCR analysis of FTO, ALKBH5, YTHDF1, and YTHDF2 transcript levels in HUVEC, Caco2, A549, and Huh7 cells at 48 hours post‑infection (MOI = 0.1, n = 3 independent experiments; error bars represent SEM. (D) Western blot analysis of METTL3, METTL14, FTO, ALKBH5, YTHDF1, YTHDF2, and YTHDF3 protein levels in Caco2 and Huh7 cells at 48 hours post‑infection (MOI = 0.1). Protein levels were normalized to GAPDH and are presented as relative expression compared to mock‑infected controls. n = 3 independent experiments; error bars represent SEM.

**Figure S4. Integrated transcriptome and m6A methylome analysis reveals co‑regulated targets in MPXV infection.** (A) GO functional enrichment analysis of intersection genes from transcriptome and m6A methylome data at 7 dpi, presented as a bar plot summarizing enriched biological processes and cellular components. (B) Scatter plot illustrating the overall correlation between changes in m6A modification levels (x‑axis, log₂FC) and changes in gene expression levels (y‑axis, log₂ FC) across three time points. (C) Quadrant plots showing correlations between gene expression change (x‑axis) and m6A modification change (y‑axis) at each time point, with selected strongly correlated genes labeled. (D) Time‑series line graphs representing transcriptional levels (red line) and m6A modification levels (blue line) for representative intersection genes over the course of infection.

**Figure S5. Schematic overview of the study workflow.** The workflow consisted of six main stages: (1) Animal Model and Experimental Design: Rhesus macaques were challenged with MPXV (10⁷ TCID₅₀, day 0, n=9) or PBS (control, n=3). Whole blood was collected at 7, 14, and 21 days post-infection (dpi). (2) Multi-Omics Profiling: RNA-seq for transcriptomics and MeRIP-seq for epitranscriptomics. (3) Bioinformatics Analysis: For RNA-seq, this included time-series expression, functional enrichment, and GSEA; for MeRIP-seq, differential modification peaks, HOMER motif, and functional enrichment analyses. (4) Immune Cell Marker Analysis: Evaluated the expression and m6A modification dynamics of lineage-specific immune markers. (5) Integrated Analysis: Combined multi-omics data to examine m6A regulator expression, link modification profiles to immune activity, and analyze pathogenic pathways (apoptosis, pyroptosis, BCL2/BRAF inhibitor pathways). (6) Validation Experiments: Performed using qRT-PCR, MeRIP-qPCR, Western blot, and ELISA.

**Figure S6. Correlation of global m6A level and m6A regulatory genes with viral load.** Scatter plots showing Spearman correlations between viral load (log₁₀ copies/mL) and (A) global m6A level, as well as (B–H) representative m6A regulatory genes across all samples (n = 12, including 3 controls and 3 per time point). ρ and p‑values are indicated; FDR‑adjusted p‑values are provided in Table S4.

**Figure S7. Cross-Omics Identifies Host m6A-Regulated Targets and Biomarkers in MPXV Infection.** (A) Temporal expression trends of key pathogenic pathway genes. Line plots showing average expression changes (log₂ fold change) of genes in six pathways (viral entry/replication, cytokine storm, apoptosis/pyroptosis, antigen presentation, interferon response, and virus-epithelium interaction) at 7, 14, and 21 dpi. (B) Temporal m6A modification trends of key pathogenic pathway genes. Line plots showing average m6A modification level changes (log₂ fold change) of genes in the six pathways at 7, 14, and 21 dpi. (C) Validation of target gene expression in colon tissue from an independent public dataset (GSE219036). Heatmap showing log₂(TPM+1) expression values of 11 genes in colon tissue, comparing Mock control against infections with West Africa, Congo, and 2022 MPXV strains. (D) Validation of target gene expression in skin tissue from an independent public dataset (GSE219036). Heatmap showing log₂(TPM+1) expression values of 11 genes in skin tissue, comparing Mock control against infections with West Africa, Congo, and 2022 MPXV strains. (E) Validation of target gene expression in lung tissue from an independent public dataset (GSE287262). Heatmap showing log₂(TPM+1) expression values of 11 genes in lung tissue, comparing Mock control against infections with Lb, Congo, and 2022 MPXV strains. (F) Validation of target gene expression in neuronal cell lines from an independent public dataset (GSE307689). Heatmap showing log₂(TPM+1) expression values of 11 genes across three neuronal cell lines (HMG3, SH-SY5Y, U87MG), comparing Control and MPXV-infected conditions.

**Figure S8-S9. Correlation of 38 persistently modified genes with viral load.** Scatter plots showing Spearman correlations between viral load and expression of 38 persistently modified genes (log₂[FPKM+1]) across all samples (n = 12). ρ and p‑values are indicated; FDR‑adjusted p‑values are provided in Table S4.

**Figure S10. Correlation of 11 candidate genes with viral load.** Scatter plots showing Spearman correlations between viral load and expression of 11 candidate genes (log₂[FPKM+1]) across all samples (n = 12). PCNA, BAX, and MCM2 showed significant positive correlations after FDR correction (ρ = 0.746, 0.746, and 0.725, respectively; all FDR < 0.05). ρ and FDR values are indicated.
